# A novel tibial tuberosity advancement technique with cranial implant fixation (TTA CF): a pilot study in sheep

**DOI:** 10.1186/s12917-018-1551-8

**Published:** 2018-08-02

**Authors:** Yauheni Zhalniarovich, Marta Mieszkowska, Paulina Przyborowska-Zhalniarovich, Joanna Głodek, Andrzej Sobolewski, Grzegorz Waluś, Zbigniew Adamiak

**Affiliations:** 1 Department of Surgery and Radiologu with Clinic, Faculty of Veterinary Medicine, University of Warmia and Mazury in Olsztyn, Oczapowskiego 14, 10-718 Olsztyn, Poland; 2 ChM sp. z o. o, Lewickie 3b, 16-061 Juchnowiec Kościelny, Poland

**Keywords:** Cranial cruciate ligament, CCL, Stifle, Osteotomy, Maquet hole, Ovine model, Dynamic stabilization

## Abstract

**Background:**

Cranial cruciate ligament rupture is the most frequent orthopedic disorder in human and animals. An array of surgical techniques have been described to stabilize the stifle joint in dogs, including intraarticular stabilization, extraarticular stabilization, and tibial osteotomy techniques. Tibial plateau leveling osteotomy and tibial tuberosity advancement with a lot of modifications are the most common. In this study we report the possibility of fixing the novel titanium implant for tibial tuberosity advancement with two screws in a craniocaudal direction. The aim of our study was to improve the clinical benefit of the tibial tuberosity advancement surgical technique where an attempt was made to reduce the number of screws and obtain a strong implant fixation with minimal bone traumatization. This way of attachment allows to distribute the forces evenly on medial and lateral side of osteotomy gap.

**Results:**

Tibial tuberosity advancement with cranial implant fixation was performed in four sheep. Complete radiographic and clinical follow up was recorded. All four sheep had a complete osteotomy line healing at a mean of 10 weeks postoperative (range 8–12 weeks). None of the animals had osteotomy gap healing grades of poor. Minor complication included the minimally cracked Maquet hole on the postoperative radiographs, without displacement of the tibial crest which occurred in 2 sheep. Major complication grade 1, major complication grade 2 and catastrophic were not found.

**Conclusions:**

The novel surgical technique for tibial tuberosity advancement with cranial implant fixation is technically comparable to the recent trend in tuberosity advancement techniques, where partial osteotomy of the cranial tibial tuberosity crest is performed. A cranial implant fixation allows to distribute the forces evenly on medial and lateral side of osteotomy gap, which may result in less number of major complications in dogs. A novel titanium implant decreases the tibial traumatisation by reducing the number of screws.

## Background

Cranial cruciate ligament (CCL) insufficiency is the most common orthopedic disorder in humans and dogs associated with pelvic limb lameness [1–4]. CCL rupture is one of the major contributors to the development of stifle joint osteoarthritis [2]. The CCL works as a primary stabilizer for both rotational and translational movements of the tibia, which is the main function to resist the cranial tibial thrust [5, 6]. Several surgical techniques have been described to stabilize the CCL ruptured stifle, including static intracapsular or static extracapsular [7–9] and dynamic techniques [10, 11]. Dynamic surgical methods provide stability during weight bearing by altering the geometry of the stifle joint [10–14]. Dynamic techniques are based on tibial osteotomies and the two most common of which are tibial plateau leveling osteotomy (TPLO) and tibial tuberosity advancement (TTA) [7].

Tibial tuberosity advancement was developed to reconstruct dynamic stability in CCL deficient stifles in dogs by reducing the patellar tendon angle (PTA). PTA is created by the tibial plateau and patella tendon [15, 16]. Stability is achieved by performing the mediolateral osteotomy and advancing the tibial tuberosity cranially [15, 16]. By changing the angle between patellar ligament and PTA to 90° during the stance phase of gait, shift forces are eliminated and the stifle is rendered dynamically stable [15]. Experimental in vitro studies attested the biomechanics of TTA [17–19], and clinical reports proved satisfactory limb function after surgery [20–23].

In the conventional TTA technique description, advancement of tibial tuberosity is achieved by using forks and cages of different sizes, which are introduced into the osteotomy line [20–24]. Cranial advancement of the tibial tuberosity varies from 3 to 12 mm depending on the cage size [15, 16]. As of 2010, > 80,000 conventional TTAs were performed by > 750 veterinary surgeons worldwide using one manufacturer’s equipment [25]. In 2014 the TTA Rapid technique was reported, which was based on the Maquet technique. In this procedure the distal cortex of the tibial crest remains intact [26].

Animal models have been widely used in orthopedic biomedical research in human and veterinary medicine [27–31]. International standards established regarding the species corresponding for testing implantation of materials in bone, state that rabbits, pigs, dogs, goats, are suitable [27, 28]. The sheep is a species frequently used in orthopedic research, because of bones size, bone and joint structure, body weight, and bone healing processes [27, 28]. The ovine model is very useful to analyze the biomechanical characteristics of bones [27, 32].

Conventional TTA and TTA Rapid are based on fixing the cage from the medial side of the tibia [16, 21, 24, 26]. In both methods there are many implants (plate, fork, cage and screws) attached to the bone. This amount of material inside the bone can cause a lot traumatization to the bone and delay regeneration. It is also can cause bone weakness or affect the strength of the forming callus. In both surgical procedures mentioned above in case of postoperative complications, such as tibial tuberosity fracture, there are K-wires drilled cranially which are stabilizing the fracture [22, 25, 45, 46]. The aim of our study was to improve the clinical benefit of the TTA Rapid surgical technique by reducing the number of screws needed to obtain strong implant fixation with minimal tibial crest traumatization and, at the same time, to prevent tibial tuberosity fracture by screw placement in aa craniocaudal plane. Moreover, the aim was to check the possibility of fixing the novel titanium implant for TTA with screws in craniocaudal direction (TTA with cranial fixation [TTA CF]) on ovine model. Thanks to the dedicated implant guide the novel titanium implant is fixed with two screws in the proximal tibia.

## Methods

### Animals

This study was performed on four female Merino sheep, skeletally mature, between 2 and 4 years of age, with body weight (BW) of 40 kg (±5 kg). The sheep were obtained through the farm with registration nr PL 050649214001 (Barczewo, Poland). The animals were kept at 15 ± 2 °C under a 12 h/12 h dark/light cycle. The animals received water ad libitum and had free access to food. Patients were monitored at all times by a specialized veterinary surgeon. All animal procedures were conducted in accordance with the Local Ethical Committee for Experiments on Animals in OLSZTYN (NR 07/2015 Olsztyn, Poland). Animals were followed up for 6 months. After 6 month observation period the sheep were euthanized by intravenous injection of pentobarbital. Each animal was kept under the Good Laboratory Practice conditions and looked after by a specialist.

### Anaesthesia

Animals were sedated with xylazine (0.1 mg/kg BW, Vetaxyl, Vet-Agro, Lublin, Poland) and general anaesthesia was induced with ketamine (3–5 mg/kg BW, Bioketan, Vetoquinol Biowet, Gorzów Wielkopolski, Poland), diazepam (0.1 mg/kg BW, Relanium, Polfa, Warsaw, Poland) and propofol (0.2–0.4 mg/kg BW, Scanofol, ScanVet, Gniezno, Poland) and maintained with intravenous administration of propofol constants rate infusion (1–5 mg/kg BW/h) with isolfurane inhalantion during the procedure (1.5–2% Aerane, Baxter, Warsaw, Poland). Carprofen (2 mg/kg BW, Carprodyl, Ceva Animal Health, Warsaw, Poland) as a painkiller and benzathine penicilline (4.5 mg/kg BW, Penicillin LA, ScanVet, Gniezno, Poland) was administered pre- and postoperatively.

### Preoperative planning

**Fig. 1:**
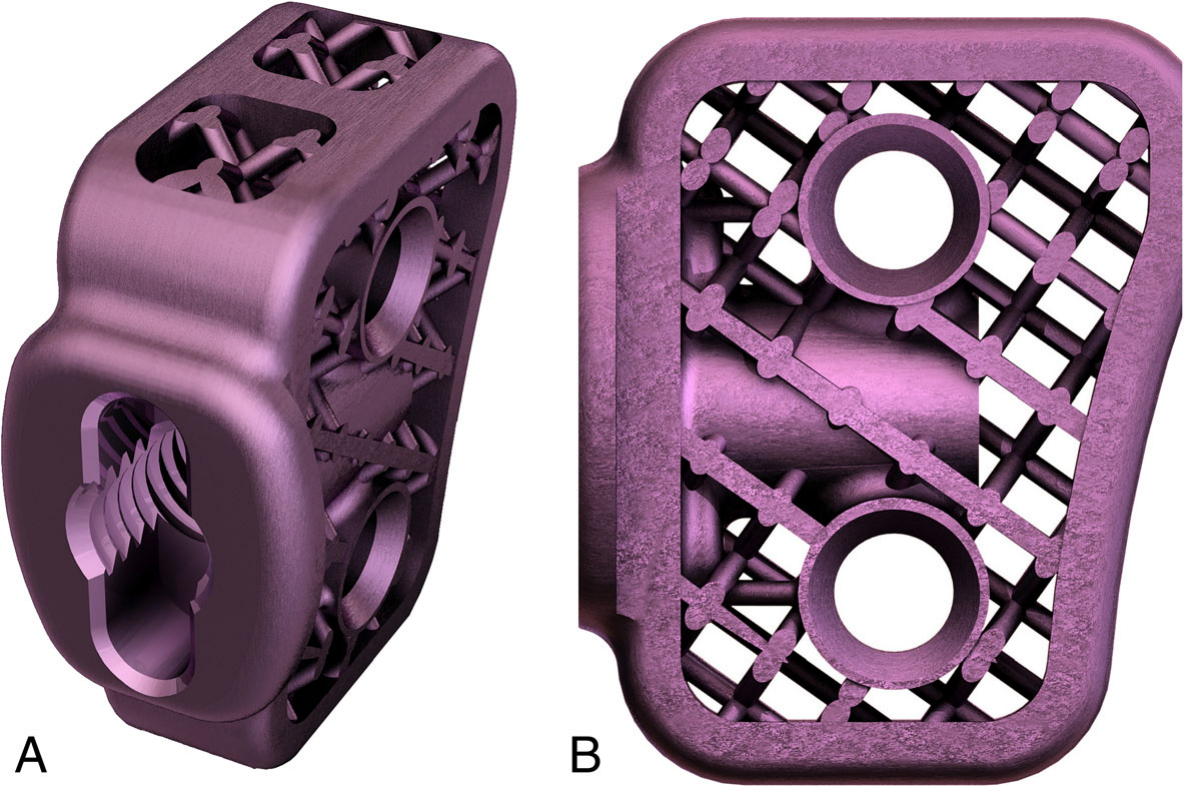
Oblique (**a**) and lateral (**b**) view of 6 mm titanium implant for TTA CF

**Fig. 2:**
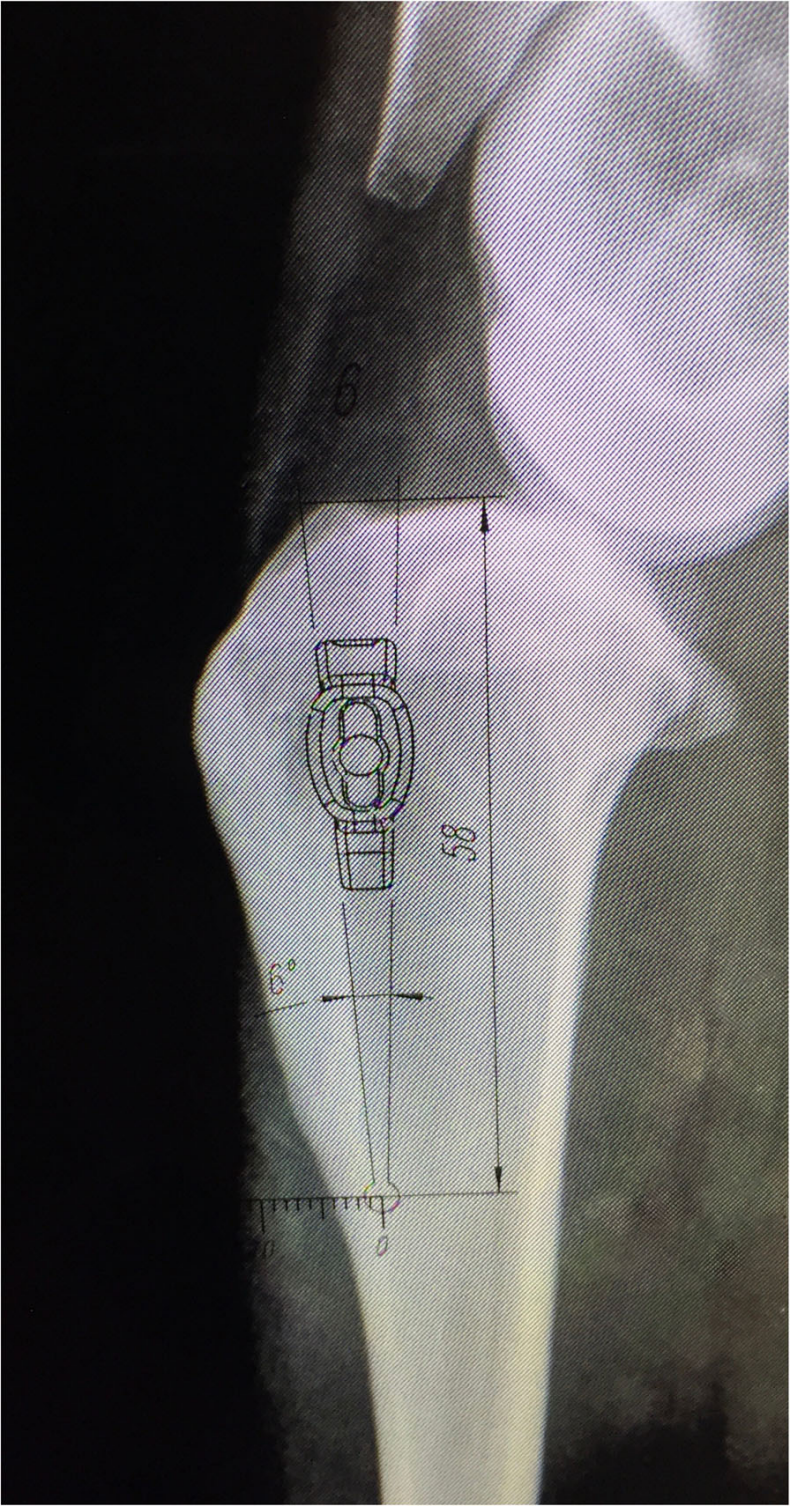
Preoperative measurements. This image shows the measurement of cranial cortex of the tibial crest using 6 mm implant template for TTA CF. With the template the exact position of the Maquet hole along long axis of the tibia is determined. In this case 4 mm cranial cortical thickness was measured

Before surgery mediolateral and craniocaudal radiographs of the stifles were performed in each sheep to assure proper advancement of tibial tuberosity. Measurements were obtained using RadiAnt DICOM software. Common tangent TTA procedure was used to assess the degree of advancement [26, 33]. In all four animals 6 mm width titanium implants were used. The proximodistal height of the implant was 20 mm and the slope of the implant was 6° (Fig. 1). Using the TTA CF template the optimal Maquet hole position, the length of the osteotomy cut, and the thickness of the cranial cortex in the region of the Maquet hole were designated (Fig. 2). Detailed information about measurements are included in Table 1.

**Table 1:** Preoperative measurements, surgery time and clinical observation

Animals	Cranial cortex thickness	Implant width	Body weight	Osteotomy length	Surgery time	Osteotomy healing time	Oservations
Sheep I	4 mm	6 mm	38 kg	58 mm	55 min	9 weeks	Minimally cracked Maquet hole on the 2 weeks postoperative radiographs
Sheep II	5 mm	6 mm	43 kg	58 mm	48 min	12 weeks	
Sheep III	5 mm	6 mm	42 kg	58 mm	40 min	11 weeks	Minimally cracked Maquet hole on the 2 weeks postoperative radiographs
Sheep IV	4 mm	6 mm	39 kg	58 mm	45 min	8 weeks	

The titanium implants were designed with porous core and produced on a 3D printer with a Selective Laser Melting (SLM) technique. The printing process takes place in a closed, sealed machine chamber. The machine chamber was filled with an inert gas - argon, which protects the surface of the produced implant against reacting with moisture coming from the powder, oxygen and other elements that can change the composition and properties of the surface layers of the printout. Therefore, the powder was in a closed powder handling system, so that it does not change its properties during the printing process. The TTA-CF implants were made of biocompatible materials which chemical composition complies with the ISO 5832–3 standard.

### Surgical procedures

The surgical technique was based on the Maquet procedure, which was firstly described in 1976 to allay pain related to osteoarthritis of the patellofemoral joint and in chondromalacia of the patella in people [34]. The sheep were located in lateral recumbency with the limb to be operated on the table with opposite limb removed from the surgery area. After the place of surgery had been shaved, the skin was disinfected completely, and a sterile surgical drape was applied.

**Fig. 3:**
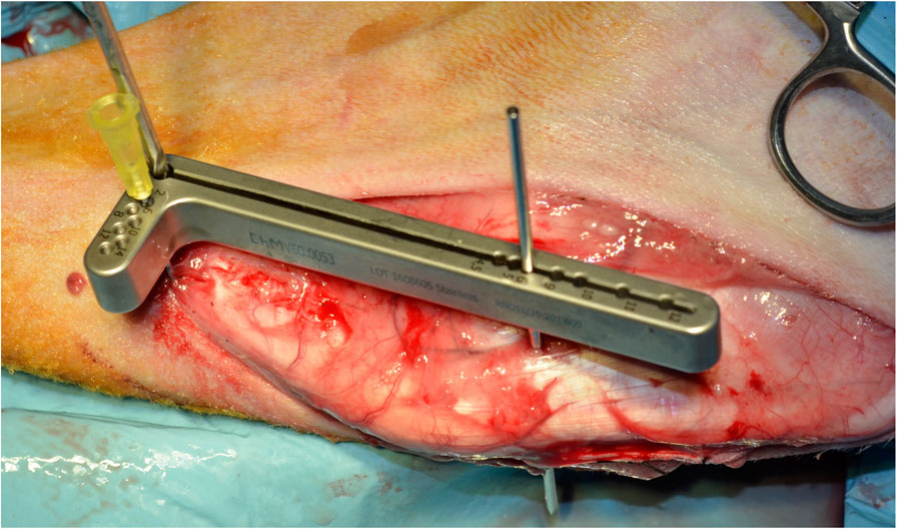
Intraoperative use of drill and saw guide for TTA CF technique. The short leg of the guide corresponded to the cranial cortical thickness of the tibial crest measured with use of template (4 mm). The long leg of the drill and saw guide has numbers corresponded to the implant number (6 mm)

**Fig. 4:**
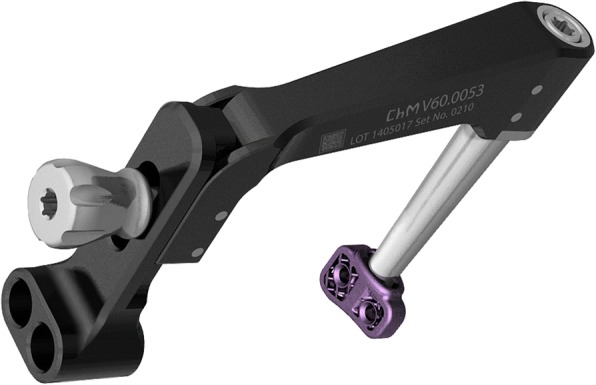
Image of the 6mmTTA CF titanium implant with a screwed dedicated implant guide

**Fig. 5:**
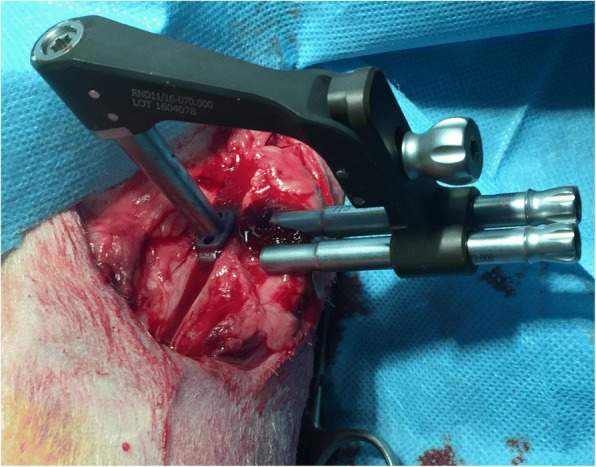
Intraoperative use of dedicated implant guide for TTA CF technique. The implant guide allows to drill 2 craniocaudal holes throught the cranial tibial crest for implant attachment

A skin incision was made from the parapatellar region to the proximal tibia on the medial side using electrocautery to control bleeding. Fasciae were transected in line with the skin incision. A medial parapatellar mini-arthrotomy was performed and LCC was intersected using a #11 scalpel blade. For tibial osteotomy the designed drill and saw guide were positioned precisely in both craniocaudal and proximodistal planes of the tibia. In proximal part a 1.2 mm K-wire was inserted perpendicular to the straight patella ligament into the distal infra patellar bursa. This K-wire was inserted into the long leg of the designed drill and saw guide. The 0.9 mm needle was inserted into the short leg of the TTA CF drill and saw guide in the hole which is corresponding to the cranial cortical thickness of the tibial crest. The Maquet hole was drilled with a 3 mm drill bit (Fig. 3). The osteotomy was performed perpendicular to the craniocaudal plane of the tibia with a 0.7 mm saw blade with the use of a TTA CF drill and saw guide. After the osteotomy was performed the drill and saw guide were removed. A 3 mm bone spreader was inserted in the osteotomy line. The spreader has an adjustment screw, by which it is possible to precisely and at the same time slightly widening the osteotomy line. The spreader has been equipped with a scale that prevents the osteotomy line from opening excessively. Gentle rotation of the adjustment screw advanced the tibial tuberosity. When the desired tuberosity advancement was achieved, the 6 mm width TTA CF implant with screwed guide was inserted into the osteotomy gap (Fig. 4). The implant guide allows to drill 2 craniocaudal holes for implant attachment (Fig. 5). The TTA CF implants were fixed with two 2.7 mm titanium self-tapping cortical screws. The implant guide was unscrewed from the implant and removed. The wound was closed in layers, starting from deep fascia with interrupted absorbable suture. Subcutaneous tissue and skin was closed with continuous absorbable material. Immediately after surgery the operated leg was radiographed in mediolateral and craniocaudal projections to evaluate the implant position. A pressure bandage was applied for 24 h.

### Radiography and clinical follow up

The operated leg was radiographed immediately postoperatively and then after every 2 weeks up to 6 months in both a craniocaudal and mediolateral projections. Positioning of the implant, position of the screws and callus formation were evaluated from the radiographs. Obtained images were evaluated by the specialized veterinary surgeon. Complete bone healing was defined when trabecular structures were visible in the osteotomy line [26]. A 5 level subjective numerical rating scale for lameness evaluation was used [31]. Lameness was graded as 0- sound lameness, 1- barely detectable, 2- mild, 3- moderate, and 4- severe lameness [31]. Postoperative complications were graded as minor, major grade 1, major grade 2 and catastrophic using a previously reported scale [35].

## Results

TTA CF with the use of a 6 mm width titanium implant was performed in four sheep. All the sheep recovered from anesthesia and surgery without complications. Post-operative.

lameness was evaluated using the previously stated subjective 5-point grading scale (from 0 to 4). During the first 7 days after surgery, we observed in some animals an obvious mild lameness (degree 2) of the left hindlimb when walking, but it improved favorably to hardly detectable lameness (degree1) during the second week [31]. The normal gait was from third week since the end of the surgical procedure (Table 2).

**Table 2:** Summary of postoperative degree of lameness

Animals/days	1 day	2 day	3 day	4 day	5 day	6 day	7 day	8 day	9 day	10 day	11 day	12 day	13 day	14 day	15 day	16 day
Sheep I	3	3	3	3	3	3	3	2	2	2	2	2	2	1	1	0
Sheep II	3	3	3	3	3	3	2	2	2	2	2	2	1	1	1	0
Sheep III	3	3	3	3	2	2	2	2	2	2	1	1	1	1	0	0
Sheep IV	3	3	3	3	3	3	2	2	2	1	1	1	0	0	0	0

Lameness was graded as norm- normal gait, 0- sound lameness, 1- barely detectable, 2- mild, 3- moderate, 4- severe lameness. Lameness was evaluated starting from 1 day after surgery

The distance of the tibial tuberosity advancement and the appropriate titanium implant size were printed based on preoperative radiographic measurements. The TTA CF implant size used was 6 mm width. The dedicated implant guide allows the surgeon to drill and fix the implant with two 2.7 mm self-tapping titanium screws craniocaudally and a 2 mm drill bit was used to perform the screw holes. A 3 mm drill bit was used to perform the Maquet hole in all patients. Complete radiographic and clinical follow up was recorded. All four sheep had a complete bone healing at a mean of 10 weeks (range 8–12 weeks). None of the animals had osteotomy gap healing grades of poor.

**Fig. 6:**
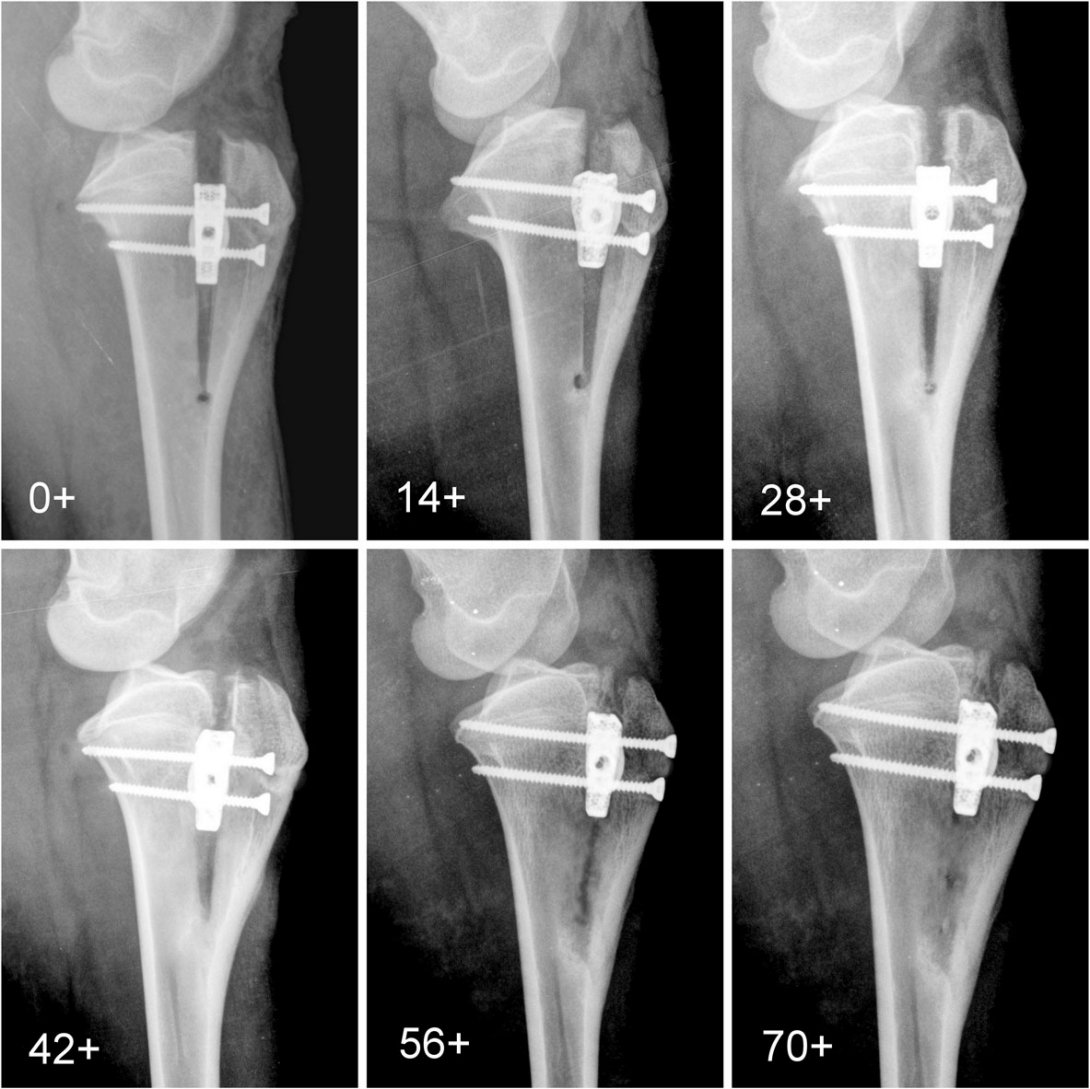
Radiographic follow up. Mediolateral radiographs of the stifle immediate postoperative and on 14, 28, 42, 56, 70 day. As a result of bone remodeling, the osteotomy gap began to disappear gradually after 42 days

After the surgery, osteotomy gaps were clearly visible. Radiographs taken during the experimental period of 6 months revealed gradual hyperplasia and progressive mineralization of bone callus at the following stages of healing (Fig. 6). Two weeks postoperatively the osteotomy line showed a clear contour. After 4 weeks small amounts of periosteal callus were observed. As a result of bone remodeling, the osteotomy gap began to disappear gradually after 6 weeks.

**Fig. 7:**
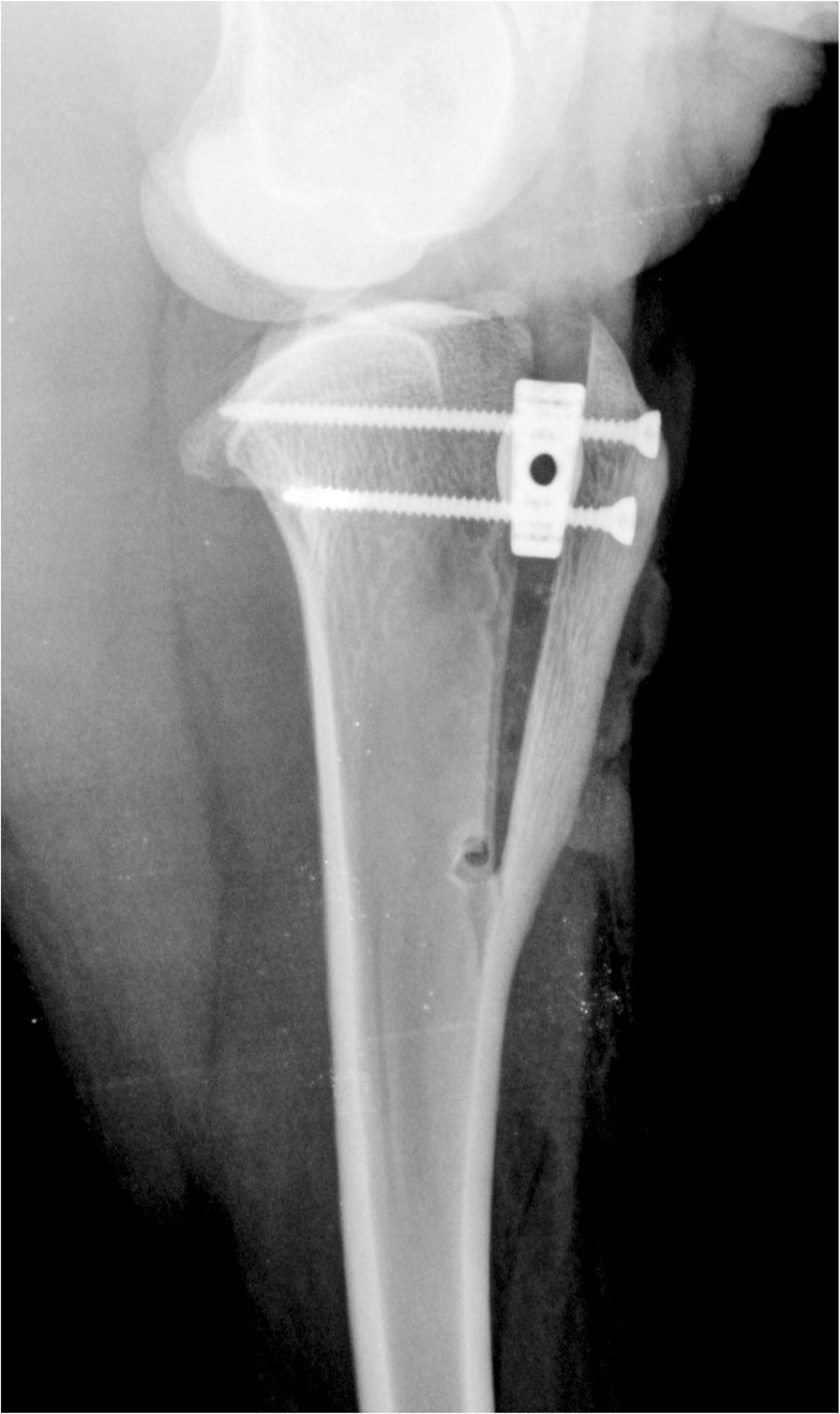
Mediolateral radiograph of the stifle. A small crack of Maquet hole occurred 14 days postoperatively

Minor complications included the minimally cracked Maquet hole on the 2 weeks postoperative radiographs, without displacement of the tibial crest occur in 2 sheep (50%) (Fig. 7). Major complication grade 1, major complication grade 2 and catastrophic were not found. None of this sheep not required any additional treatment.

## Discussion

Rupture of CCL is the most common orthopedic disorder [1–4]. The CCL stabilize both rotational and translational movements of the tibia [36, 37]. There are two common types of TTA procedure in dogs [15, 26]. TTA restore dynamic stability in CCL ruptured stifles by reducing the angle formed by the tibial plateau and the simple patellar ligament through the osteotomy of the tibial tuberosity in the dorsal plane and advancing the tibial crest. The dimension of advancement is based on the preoperative measurements of patellar ligament and plateau angle [15, 16, 22]. The TTA surgical technique is based on the Maquet procedure, which was presented to allay pain related to osteoarthritis of the patellofemoral joint and in chondromalacia of the patella in people [34]. Our modified TTA CF technique is a modification of conventional TTA and TTA Rapid technique, where an attempt is made to reduce the number of screws and obtain a strong implant fixation only with two screws in craniocaudal direction. In the current experiment, TTA with cranial implant fixation was investigated.

In the last two decades, conventional TTA and TTA Rapid have become a successful and respected surgical techniques for CCL insufficiency treatment [21, 22, 25, 26, 37, 38]. Both techniques have some limitations. In conventional TTA the surgeon has to ensure sufficient bone stock on the cranial part of tibial crest and sufficient space on the tibial shaft to allow attachment of the fork, plate and bone screws [22]. In TTA Rapid technique the cage is attached only on one side, all the screws are mounted from the medial side of the tibia. Therefore, mobility remains on the lateral surface of the osteotomy gap. This report describes the TTA CF technique, where the implant is attached with two 2.7 mm screws in a craniocaudal direction. The designed implant guide allows for the introduction of two screws through the center of cranial tibial crest.

We have reported the TTA CF technique on ovine model. Sheep are becoming popular as animal model in orthopedic research in both human and veterinary medicine [28, 32, 39, 40, 42]. In humans, the use dogs are reduced due to the emotional impact related to these kinds of animals [28]. Sheep were used to study repair of fractures and articular ligaments, limb lengthening, and treatment of osteoporosis and osteoarthrosis [28]. TTA CF titanium implants and special guides are dedicated for dogs affected by CCL insufficiency. The sheep was chosen as a model animal because the anatomy and biomechanics of the stifle joint are similar to dogs [28, 32, 39, 40, 42].

Sheep reach sexual maturity between seven and 12 months of age. At sexual maturity, the animals are not mature from skeletal point of view yet. The proximal tibial ossification center closes between 20 and 26 months of age, and the distal tibia closes between 12 and 18 months. This report has been carried out on four female Merino sheep, skeletally mature between 2 and 4 years of age.

The TTA CF surgical technique is based on the Maquet procedure and has been derived from descriptions of the procedure in man [34]. With this surgical procedure in dogs, the osteotomy is performed in the frontal plane and left incomplete, so the tibial crest retains its distal attachment to the tibia [26, 41]. Once the osteotomy is made, the cranial tibial crest is advanced and held in position with implant [26, 41]. Our novel attachment method and the appropriate implant guide allow the firm fixation of the implant. Fixing the screws in a craniocaudal direction allows to distribute the forces evenly on medial and lateral side of osteotomy gap.

All Maquet holes were created with a 3 mm drill bit. In previous reports the diameter used for Maquet hole drilling varied from 2 to 3 mm [26, 43, 44]. A 3 mm hole allows for better force distribution throughout the osteotomy line [43], but on the other hand it creates a larger bone defect [26]. Because the size of implant is often related to the size of animal, a 2 mm and 2.5 mm drill bit can be used in animals under 20 kg of BW.

We report the minor complication occurred in 2 sheep. The minor complication included the minimally cracked Maquet hole on the 2 weeks postoperative radiographs (Fig. 7). No major complication was noted. The TTA Rapid has an overall complication rate of 34%, based on limited case numbers [26]. Patellar ligament thickened and tibial fractures have been met most frequently at the level of distal cortical hinge [26]. Postoperative complications associated with conventional TTA technique include implant failure (0.8–2%), tibial fractures (1–5.1%), patellar luxation (0.8–1%) and infections (0.5–5.3%) [22, 25, 45, 46]. The presented TTA CF technique has the task to allow the firm fixation of the implant and prevent tibial tuberosity fractures.

Our report has some limitations. Experience of the surgeon was not taken into account. All TTA CF surgeries were performed by an experienced TTA surgeon. The number and type of complication might be higher or different with less experienced surgeon. Lameness evaluation was scored using a subjective scale of 0–4. Force plate analysis, would have been perfect, but was not available at the authors’ institution. Further studies are needed to determine if the results remain the same in dogs and what kind of complications will occur. Another limitation is that post TTA patellar desmopathy has not been evaluated. Although the long time radiographic follow-up is needed to determine the progression of osteoarthritis of stifle joint, however this was not the objective in our study.

The novel surgical technique for tibial tuberosity advancement with cranial implant fixation is technically comparable to the recent trend in TTA, where partial osteotomy of the cranial tibial tuberosity crest is performed [26, 41, 47–49]. Accurate preoperative osteotomy planning is critical to achieve the perfect and good outcomes. TTA CF implant is fixing in tibia with two screws in craniocaudal direction, allowing to distribute the forces evenly on medial and lateral side of osteotomy gap. In addition, the novel titanium implant decreases the tibial traumatization by reducing the number of screws.

## Conclusions

Our novel tibial tuberosity advancement technique with cranial fixation (TTA CF) is a modification of conventional TTA and TTA Rapid technique, where an attempt is made to reduce the number of screws and obtain a strong implant fixation only with two screws in craniocaudal direction. A cranial implant fixation allows to split the forces evenly on medial and lateral side of osteotomy gap, which may result in less number of major complications in dogs. A novel titanium implant decreases the tibial traumatisation by reducing the number of screws. Therefore this technique may be advantageous for surgeons and patients.

## Abbreviations

BW: Body weight
CCL: Cranial cruciate ligament
h: Hour
kg: Kilogram
NR: Number, mg- milligram
PTA: Patellar tendon angle
TPLO: Tibial plateau leveling osteotomy
TTA CF: Tibial tuberosity advancement with cranial fixation
TTA: Tibial tuberosity advancement
